# DNA Methylation Profiles of Airway Epithelial Cells and PBMCs from Healthy, Atopic and Asthmatic Children

**DOI:** 10.1371/journal.pone.0044213

**Published:** 2012-09-06

**Authors:** Dorota Stefanowicz, Tillie-Louise Hackett, Farshid S. Garmaroudi, Oliver P. Günther, Sarah Neumann, Erika N. Sutanto, Kak-Ming Ling, Michael S. Kobor, Anthony Kicic, Stephen M. Stick, Peter D. Paré, Darryl A. Knight

**Affiliations:** 1 James Hogg Research Centre at the Heart and Lung Institute, Department of Medicine, University of British Columbia and St. Paul’s Hospital, Vancouver, British Columbia, Canada; 2 Department of Anesthesiology, Pharmacology and Therapeutics, University of British Columbia, Vancouver, British Columbia, Canada; 3 Prevention of Organ Failure Centre of Excellence, Vancouver, British Columbia, Canada; 4 Department of Medical Genetics Centre for Molecular Medicine and Therapeutics, Child and Family Research Institute, University of British Columbia, Vancouver, British Columbia, Canada; 5 Department of Respiratory Medicine, Princess Margaret Hospital for Children, Perth, Western Australia, Australia; 6 School of Paediatrics and Child Health, University of Western Australia, Nedlands, Western Australia, Australia; 7 Telethon Institute for Child Health Research and Centre for Child Health Research, University of Western Australia, Nedlands, Western Australia, Australia; 8 Respiratory Division, Department of Medicine, University of British Columbia, Vancouver, British Columbia, Canada; Geisel School of Medicine at Dartmouth, United States of America

## Abstract

**Background:**

Allergic inflammation is commonly observed in a number of conditions that are associated with atopy including asthma, eczema and rhinitis. However, the genetic, environmental or epigenetic factors involved in these conditions are likely to be different. Epigenetic modifications, such as DNA methylation, can be influenced by the environment and result in changes to gene expression.

**Objectives:**

To characterize the DNA methylation pattern of airway epithelial cells (AECs) compared to peripheral blood mononuclear cells (PBMCs) and to discern differences in methylation within each cell type amongst healthy, atopic and asthmatic subjects.

**Methods:**

PBMCs and AECs from bronchial brushings were obtained from children undergoing elective surgery for non-respiratory conditions. The children were categorized as atopic, atopic asthmatic, non-atopic asthmatic or healthy controls. Extracted DNA was bisulfite treated and 1505 CpG loci across 807 genes were analyzed using the Illumina GoldenGate Methylation Cancer Panel I. Gene expression for a subset of genes was performed using RT-PCR.

**Results:**

We demonstrate a signature set of CpG sites that are differentially methylated in AECs as compared to PBMCs regardless of disease phenotype. Of these, 13 CpG sites were specific to healthy controls, 8 sites were only found in atopics, and 6 CpGs were unique to asthmatics. We found no differences in the methylation status of PBMCs between disease phenotypes. In AECs derived from asthmatics compared to atopics, 8 differentially methylated sites were identified including CpGs in *STAT5A* and *CRIP1*. We demonstrate STAT5A gene expression is decreased whereas CRIP1 gene expression is elevated in the AECs from asthmatic compared to both healthy and atopic subjects.

**Discussion:**

We characterized a cell specific DNA methylation signature for AECs compared to PBMCs regardless of asthmatic or atopic status. Our data highlight the importance of understanding DNA methylation in the epithelium when studying the epithelial contribution to asthma.

## Introduction

Asthma and atopy are two of the most common chronic inflammatory conditions in the western world [1]. While atopy affects up to half of the adult population, only 20% of these patients develop asthma [1]. Although the allergic inflammatory mechanisms in both diseases are similar, it is clear that genetic, environmental and epigenetic factors are important in the pathogenesis of atopy and asthma. Many studies have investigated the role of both environmental and genetic factors in the development of atopy and asthma however there is little understanding of the epigenetic impact.

Epigenetic control of gene expression has two general mechanisms: either the DNA itself is chemically altered by the addition of a methyl group to a cytosine base in a cytosine-guanine (CpG) dinucleotide by a DNA methyltransferase (DNMT), or the histone proteins that package DNA into chromatin are chemically modified. DNA methylation generally results in gene silencing or repression by either changing the conformation of chromatin (facilitated by methyl CpG binding domain proteins (MBDs) and histone deacetylases), or by simply blocking transcriptional machinery from accessing the DNA. Thus, although the genetic make-up of an individual is identical throughout different tissues of the body, DNA methylation is important for the regulation of unique gene expression profiles in each tissue or cell type [2].

Epigenetic modifications to DNA have been described as the potential link between environmental effects and clinical phenotypes [3]. For example, in genetically identical individuals phenotypic discordance does not exist in early life, however environmental exposures throughout the individuals life result in epigenetic disparity, which influence factors such as disease susceptibility [2]. Hollingsworth *et al*., have demonstrated that epigenetic modifications can also be inherited by using a murine model in which a maternal diet, rich in methyl donors, was shown to increase the severity of allergic airway disease in offspring [4]. Furthermore, this effect was demonstrated to be inherited transgenerationally as offspring of male pups (which were exposed to a high methyl diet *in utero*) also had increased severity of allergic airway disease [4].

The tracheo-bronchial epithelium is the interface between the environment and the submucosa and represents the first line of defense against inhaled exogenous agents. There is now substantial evidence that the airway epithelium of subjects who have asthma is abnormal, including increased expression of epithelial growth factor receptor (EGFR) at denuded and damaged sites [5], [6], [7], [8], [9], altered expression of adhesion proteins E-cadherin and zonula occluden-1 [10] and elevated numbers of basal cells marked by cytokeratin-5 [11], [12]. Cultured asthmatic epithelial cells have also been shown to be more vulnerable to oxidant-induced stress and display aberrant expression of heat-shock proteins and proinflammatory transcription factors [13], [14], [15], [16], [17]. Thus, it is important to identify if airway epithelial-specific DNA methylation patterns are altered with diseased epithelium in order to fully understand the disease process. To allow us to understand the methylation signature of the airway epithelium, we compared DNA methylation of airway epithelial cells (AECs) with peripheral blood mononuclear cells (PBMCs), as they are a minimally invasive and readily available source of biological material.

The objectives of this study were first to identify the DNA methylation signature of AECs compared to PBMCs. The second objective was to identify epigenetic differences within AECs and PBMCs from non-atopic asthmatic, atopic asthmatic, atopic and healthy children.

## Methods

### Subjects and Ethics Statement

This study is part of ongoing research investigating the phenotype of AECs from mild asthmatic and atopic children [14]. Initially, 25 children undergoing elective surgery for non-respiratory conditions were recruited as part of the DNA methylation study. For validation, the next 44 children were recruited in sequence as part of the gene expression study. Children were characterized as healthy non-atopic non-asthmatic, atopic non-asthmatic, non-atopic asthmatic and atopic asthmatic. Asthma was defined as physician-diagnosed asthma plus documented wheeze by a physician in the past 12 months. Positive responses to relevant questions on the ISAAC [18] and ATS [19] respiratory questionnaires as reported by the parents or subject were used to corroborate the diagnoses and negative responses were used to validate absence of respiratory symptoms. Atopic status was determined by positive RAST or skin prick tests to common allergens (e.g. grass pollens, mold, animal dander, peanut, house dust mite). The recruited children had no record of any infections in the 3 months prior to surgery and were not taking glucocorticoids. All parents/legal guardians gave written informed consent for this study, which was approved by the Princess Margaret Hospital for Children Ethics Committee. Subject demographics are presented in Table 1 for the DNA methylation group and Table 2 for the gene expression group. The use of human AECs and PBMCs was approved by the Providence Health Care Research Ethics Committee of the University of British Columbia.

**Table 1:** Patient demographics in DNA methylation cohort prior to surgery.

Phenotype	Number	Sex (M/F)	Average Age (range)
Healthy	7	4/3	7.28 (4.6–10.1)
Atopic	9	8/1	6.78 (4.5–10.9)
Atopic Asthmatic	4	4/0	8.4 (3.3–14.6)
Non-Atopic Asthmatic	5	4/1	5.96 (2.4–10.5)

Subjects are identified as healthy, atopic, atopic asthmatic, or non-atopic asthmatic.

**Table 2:** Patient demographics in gene expression cohort prior to surgery.

Phenotype	Number	Sex (M/F)	Average Age (range)
Healthy	15	6/9	5.07 (1.2–12.9)
Atopic	14	7/7	7.86 (2.2–16.5)
Atopic Asthmatic	15	8/7	7.74 (1.3–14.1)

Subjects are identified as healthy, atopic, or atopic asthmatic.

### AEC and PBMC Isolation

AECs were obtained by either trans-laryngeal, non bronchoscope brushing [14], [20] or via a portable ‘bronchoscope directed’ sampling methodology [21]. Briefly, children were anaesthetized and an unsheathed nylon cytology brush was used to gently rub against the epithelial surface. Once removed, the brush tip was cut off and placed into 5mL of collection media and vortexed to release cells. AECs were grown in Bronchial Epithelial Growth Medium (BEGM, LONZA, Walkersville, MD) containing 100U/mL penicillin and 100 ug/mL streptomycin, at 37°C in a humidified 95% air/5% CO_2_ atmosphere culture and collected at passage 2 and 3. PBMCs were isolated from 10 mL of whole blood using ficoll gradient centrifugation as previously described [22], [23]. DNA from AECs and PBMCs was extracted from 1×10^6^ cells using the Flexi Gene DNA Kit and QIAamp columns according to the manufacturer’s instructions (Qiagen, ON). Total RNA was extracted from AECs using the RNeasy mini kit (Qiagen).

**Figure 1:**
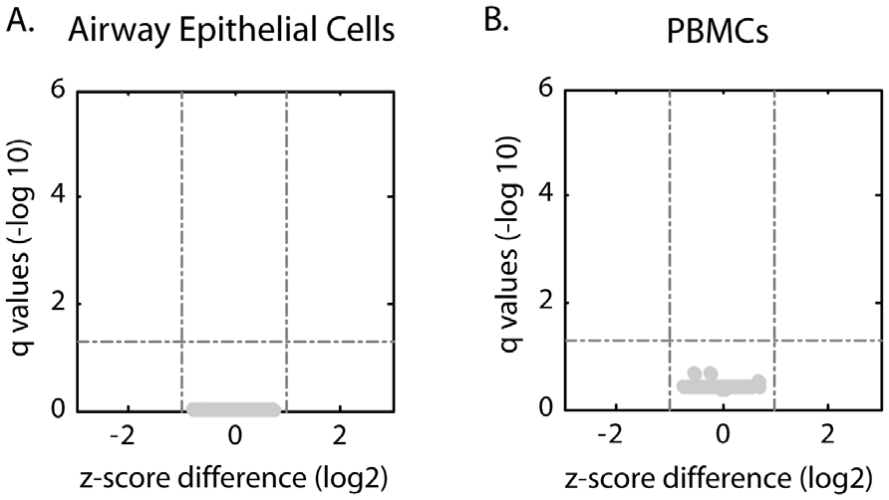
DNA methylation analysis of atopic asthmatics compared to non-atopic asthmatics individuals in AECs and PBMCs. Volcano plots of airway epithelial cells (AECs) (A) and PBMCs (B), analyzed for 1027 CpG loci in 671 genes, from atopic and non-atopic asthmatic subjects. Y-axis represents the q-values (−log_10_) for all of the CpG sites analyzed and the x-axis is the z-score difference (log_2_). Dashed lines indicate cut-offs for significance. These results show no differences between atopic and non-atopic asthma.

### DNA Bisulfite Conversion and Methylation Assay

Bisulfite treatment of 250 ng of genomic DNA was performed using the EZ DNA Methylation-Gold Kit (Zymo Research, Orange, CA). DNA methylation of 1505 CpG sites across 807 genes were analyzed using the Illumina GoldenGate Methylation Cancer Panel I, according to the manufacturer’s protocol (Illumina, San Diego, CA). The GoldenGate Methylation Cancer Panel I has previously been validated with other technologies such as pyrosequencing [24] and methylation-specific PCR [25], [26]. We use the target ID which is a locus specific identifier to refer to CpG sites. This ID contains information about the gene, distance to transcription start site, and the reference strand (e.g. STAT5A_E42_F).

**Figure 2:**
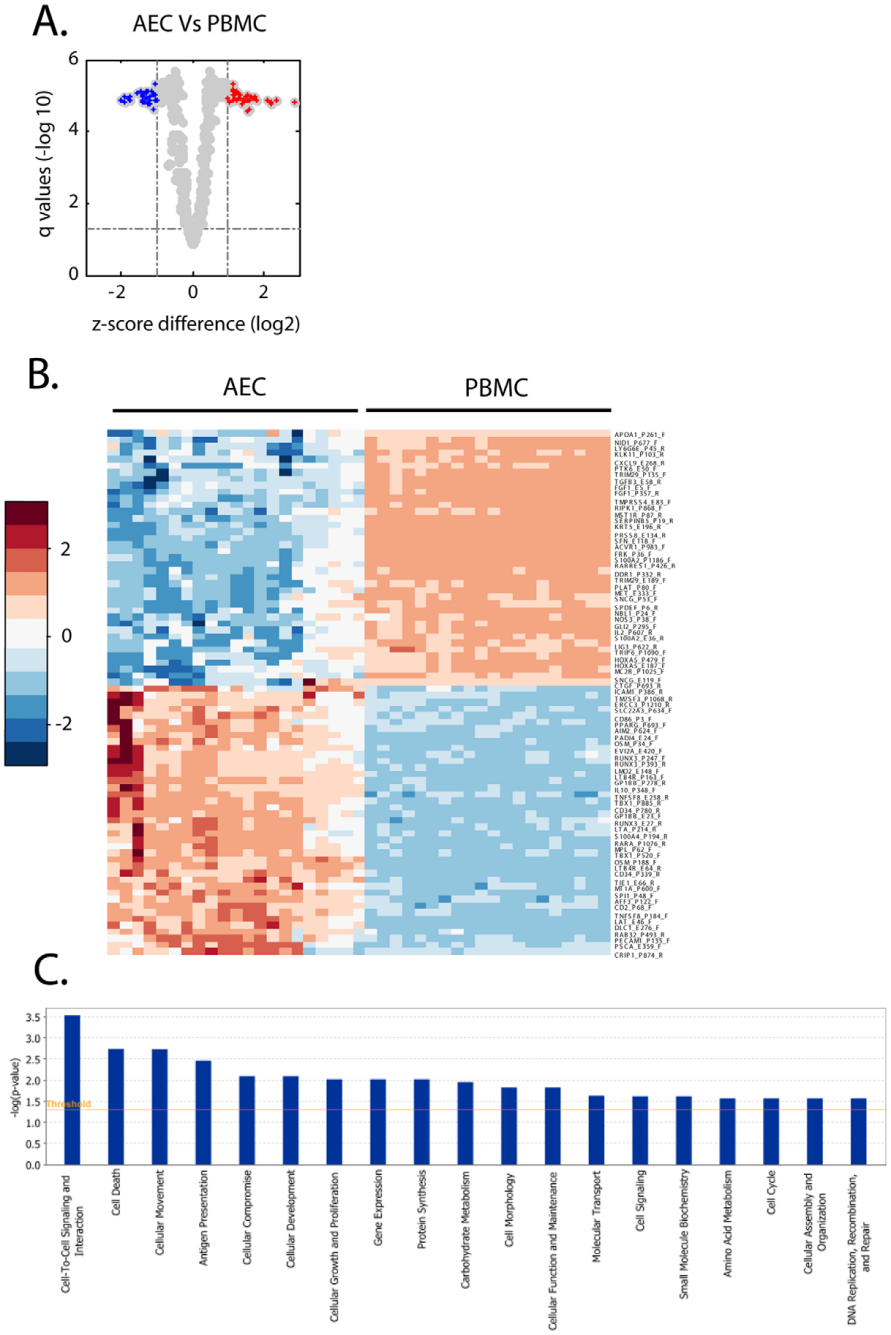
DNA methylation profile of AECs compared to PBMCs. DNA methylation for 1027 CpG sites was assessed in AECs compared to PBMCs from all subjects. A. Volcano plot of CpG sites interrogated with red and blue points indicating significantly over- and under-methylated sites. Q-values are shown on the y-axis (−log_10_) and z-score difference on the x-axis (log_2_). Dashed lines indicate cut-offs for significance. B. Heatmap illustrating z-scores of 80 differentially methylated loci in AECs compared to PBMCs. Columns represent subjects and rows represent CpG sites while red/blue indicates more/less methylated. C. The molecular and cellular functions of the 67 genes classified by IPA. The x-axis shows functions while the y-axis shows –log(p-value).

### Statistical Analysis of DNA Methylation Data

A Kruskal-Wallis test with a Dunn’s Multiple Comparison test between disease phenotype groups for sex (p = 0.22) and age (p = 0.84) and found no significant differences thus we did not adjust for these parameters in subsequent analyses. To avoid gender bias, all CpG sites on the X chromosome (n = 84) were removed from the analysis as well as probes containing single nucleotide polymorphisms (n = 272). Probes with detection *P*-values >0.05 in more than 10% of samples (n = 122) were also removed leaving 1027 CpG sites across 671 genes. Remaining loci with detection *P*-values >0.05 were imputed using the K-nearest neighbour algorithm (0.5% of data points) [27]. Raw data was then background corrected and adjusted for color bias using the Bioconductor *methylumi* package [28] and batch effects using distance weighted discrimination [29]. For each sample, the M-value: M = log_2_ ((Cy5+1)/(Cy3+1)) was calculated, where Cy5 and Cy3 were the methylated and unmethylated sequences, respectively [30]. These data were then standardized by z-score transformation. Briefly, each value was standardized by subtracting the sample mean and then dividing by the sample standard deviation [31]. Differentially methylated sites between two groups (PBMCs versus AECs and phenotypic categories) were determined using a *t*-test [32]. The false discovery rate (FDR) of differentially expressed sites between two subject categories was then calculated [33] and used to control for multiple comparisons. Restrictions of 5% q-value and a z-score difference of 2 were employed to limit data to the most relevant results. Batch effect correction, z-score transformation, and t-test computations were performed in MATLAB (MathWorks Inc., MA). All preprocessing was performed in R version 2.12.0 (http://www.R-project.org) [34]. Raw data and normalized data have been submitted to the Gene Expression Omnibus database accession # GSE37853.

**Figure 3:**
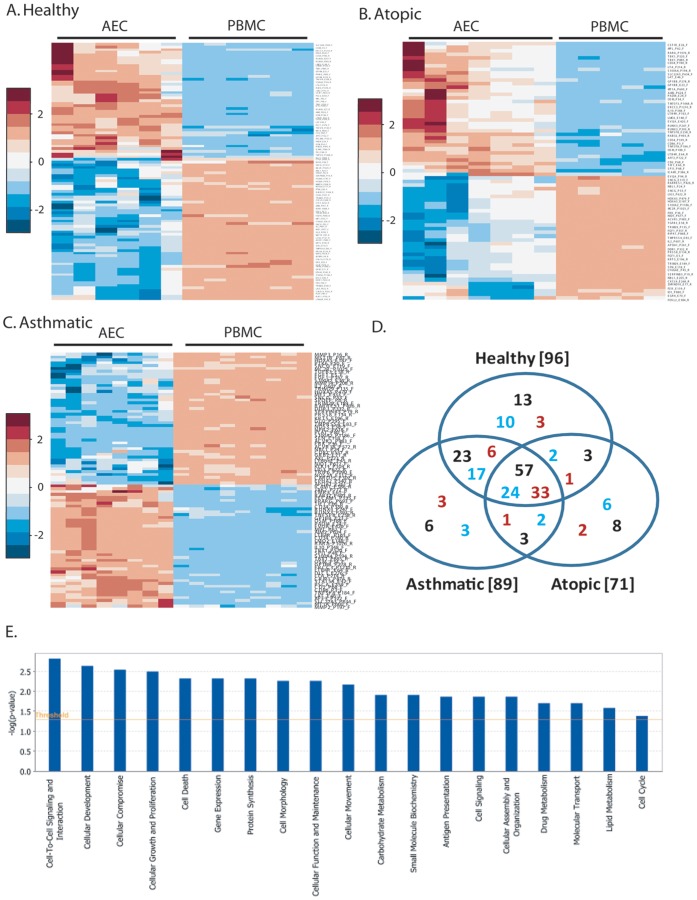
DNA methylation heatmaps of CpG sites in PBMCs and AECs from healthy, atopic and asthmatic pediatric subjects. AECs and PBMCs were analyzed for 1027 CpG loci in 671 genes from healthy (A), atopic (B), and asthmatic (C) subjects. Heatmaps of z-scores for AECs and PBMCs are shown with individuals (columns) and differential CpG sites (rows). Increased methylation is shown in red and decreased methylation in blue. D. Venn diagram showing overlap of differentially methylated sites between healthy, atopic and asthmatic subjects. Numbers in black indicate total number of CpG sites while numbers in red/blue indicate more/less methylated in AECs (compared to PBMCs). E. The molecular and cellular functions in the 47 genes classified by IPA. The x-axis shows functions while the y-axis shows –log(p-value).

### Ingenuity Pathways Analysis (IPA)

The genes containing CpG sites which showed differential DNA methylation between AECs and PBMCs were classified by IPA (Ingenuity® Systems, www.ingenuity.com) using the biological functions application. As the Illumina GoldenGate Cancer Panel I is an array based on cancer related genes, we used our dataset as the reference set for pathways analysis to avoid bias towards cancer associated pathways. A right-tailed Fisher’s exact test was used to calculate a *P*-value which determined if the association between the genes identified and the biological pathway was a result of chance alone.

**Figure 4:**
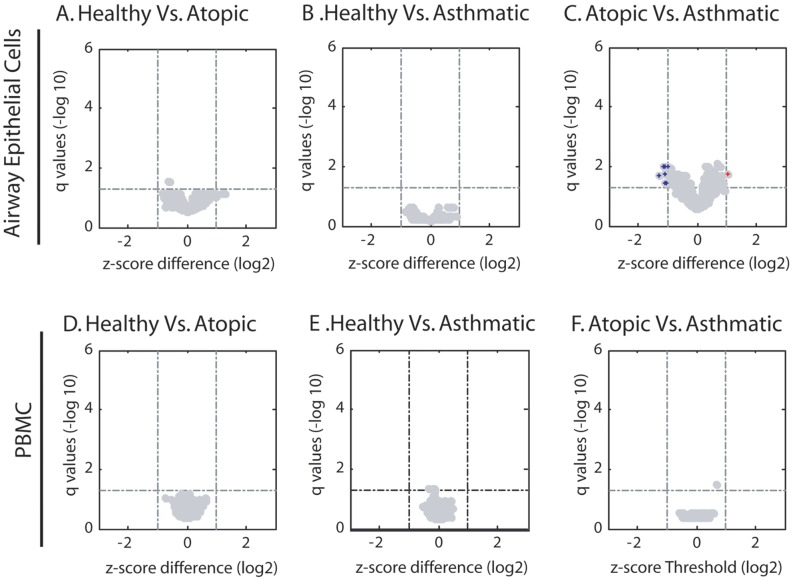
Differential methylation between disease phenotypes in AECs or PBMCs. Volcano plots of CpG sites interrogated with red and blue points indicating significantly over- and under-methylated sites. Q-values are shown on the y-axis (−log_10_) and z-score difference on the x-axis (log_2_). Dashed lines indicate cut-offs for significance. Within AECs, differences in DNA methylation were assessed in healthy subjects compared to atopics (A) and asthmatics (B) as well as atopic subjects compared to asthmatics (C). The same comparisons were performed in PBMCs (D, E, and F).

### Quantitative Polymerase Chain Reaction

Gene expression of cysteine-rich protein 1 (CRIP1) and signal transducer and activator of transcription 5A (STAT5A) was determined via two-step reverse transcriptase polymerase chain reactions (RT-PCR). Briefly, cDNA was synthesized using radom hexamanucleotide primers and Multiscribe Reverse Transcriptase (Applied Biosystems) and used in a PCR reaction containing 2X Sybrgreen Master Mix (Applied Biosystems) under standard cycling conditions. Primers for CRIP1 (*forward*: 5′-CGGAGCCGTCATGCCCAAGT-3′, *reverse* 3′-CCGATGCCAGTCCTTGCCCA-5′), STAT5A (*forward*: 5′-CCTGGACTTTTCTGAAGGGGCTCA-3′, *reverse* 3′-ATCCCGGGCTCTGGAAATCCCA-5′) and PPIA (*forward*: 5′-TGAGCACTGGAGAGAAAGGA-3′, *reverse*: 3′-CCATTATGGCGTGTAAAGTCA-5′) were obtained from GeneWorks (Hindmarsh, Australia). Expression was normalized to PPIA. A Kruskal-Wallis and a *post hoc* Dunn’s multiple comparison test was performed using GraphPad Prism Version 4.0 for Windows (GraphPad Software, San Diego California USA, www.graphpad.com). A p-value of less than 0.05 was deemed significant.

**Table 3:** Differentially Methylated CpGs in Atopic Compared to Asthmatic Derived AECs. Z-score difference is presented as atopic relative to asthmatic derived AECs.

CpG Site	Protein Function	z-score difference (log2)	q-value
CRIP1_P874_R	LIM domain protein involved in cell adhesion and differentiation	−1.14	0.01
FGFR1_P204_F	Growth factor receptor involved in mitogenesis and differentiation	−1.11	0.01
STAT5A_E42_F	Transcriptional activator involved in cell differentiation and proliferation	−1.01	0.01
S100A2_P1186_F	Calcium binding protein involved in cell cycle progression and differentiation	1.03	0.02
ITGA2_P26_R	Integrin involved in cell adhesion	−1.07	0.02
EGR4_E70_F	Transcription factor involved in mitogenesis and differentiation	−1.31	0.02
ID1_P880_F	Transcriptional inhibitor involved in cell growth and senescence	−1.03	0.04
IGSF4C_E65_F	Cell adhesion protein	−1.09	0.04

## Results

### Cell-specific Methylation Profiles in AECs Compared to PBMCs

We first determined whether there were any differences in the epigenetic signatures of PBMCs and AECs from non-atopic and atopic asthmatic subjects. As demonstrated in Figure 1, we found no difference in the DNA methylation signatures between atopic asthmatics and non-atopic asthmatics for any of the CpG sites interrogated in either AECs (Figure 1A) or PBMCs (Figure 1B). Thus although our sample size is small, for the population used, atopic asthmatic and non-atopic asthmatic subjects were grouped for subsequent analyses.

To determine if a cell specific DNA methylation profile could be obtained from patient-matched PBMCs and AECs, we initially compared the DNA methylation status of all 1027 CpG loci across 671 genes between PBMCs and AECs from all individuals (n = 25). We were able to construct a cell specific signature consisting of 80 CpG sites across 67 genes which had differential DNA methylation in AECs compared to PBMCs (Figure 2A and 2B). Table S1 contains a list of all CpG sites identified, the Z-score difference and q-value. These genes were classified by IPA which identified 19 biological functions including cell-to-cell signaling and interaction, cell death, cellular movement, antigen presentation, and cellular compromise (Figure 2C). Table S2 shows the 67 annotated genes involved in each biological function.

**Figure 5:**
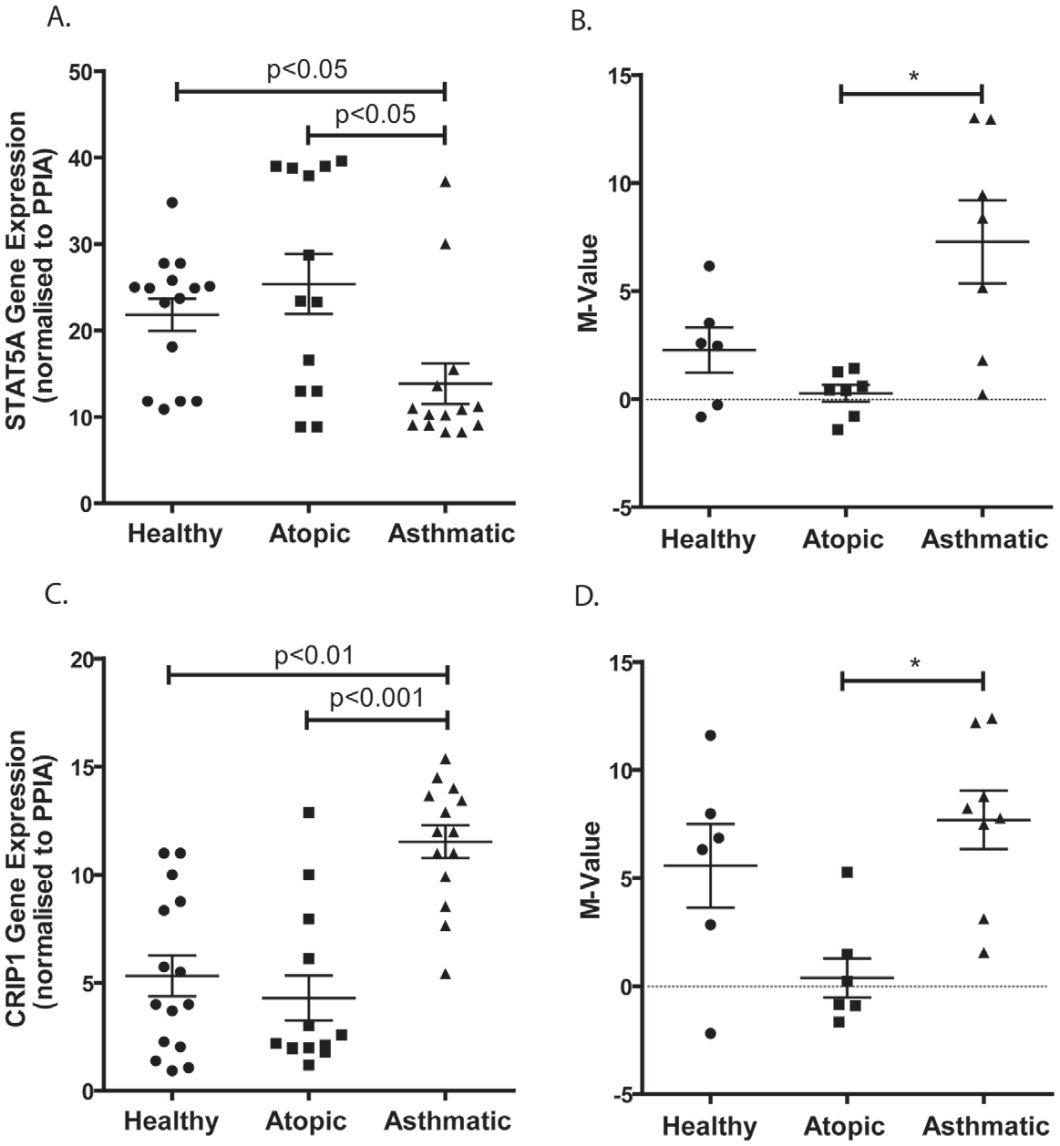
STAT5A and CRIP1 Gene Expression and DNA Methylation Status in AECs. AECs from atopic, healthy and asthmatic individuals were analyzed for STAT5A (A) and CRIP1 (C) mRNA expression using RT-PCR. Results are expressed as gene expression normalized to PPIA (y-axis). DNA methylation status is shown as M-values for STAT5A_E42_F (B) and CRIP1_P874_R (D) for the three phenotypes. * indicates differential methylation as detailed in Table 3.

We then evaluated the DNA methylation profiles of PBMCs and AECs for each disease phenotype and found 96, 71, and 89 differentially methylated CpG sites across 83, 58, and 77 genes in the healthy, atopic and asthmatic groups respectively (Figure 3A, B, and C). A full list of differentially methylated CpG sites for each disease phenotype can be found in the supplementary data online (Tables S3, S4, and S5). Many of the CpG sites identified were similar between the three phenotypes as detailed in the Venn diagram demonstrating overlap between each group (Figure 3D). This analysis also identified CpG sites that are differentially methylated between AECs and PBMCs that were unique for each phenotype; 6 for asthmatics, 8 for atopics, and 13 for healthy controls. For all individuals, 57 CpG sites across 47 genes from the original 80 CpG sites were differentially methylated in AECs compared to PBMCs irrespective of disease. The IPA analysis of these AEC specific genes identified 19 overrepresented biological and molecular functions, the top three being cell-to-cell signaling and interaction, cellular development, and cellular compromise as detailed in Figure 3E and Table S6.

### Comparison of DNA Methylation Profiles of AECs or PBMCs between Disease Phenotypes

We next sought to determine if there were differences in the DNA methylation profiles of AECs from asthmatics compared to healthy controls. We found no differentially methylated CpG sites in this comparison (Figure 4A). We also analyzed differences in DNA methylation between atopic and healthy subjects and again found no differentially methylated CpG sites (Figure 4B). As only a portion of atopic individuals develop symptoms of asthma, we next examined whether this dichotomy was associated with differential DNA methylation status of AECs in these two cohorts. Comparison of AECs from asthmatic and atopic children showed eight differentially methylated CpG sites from eight different genes (Figure 4C). The q-values and Z-score differences for these eight differentially methylated CpG sites are presented in Table 3.

When the DNA methylation signatures of PBMCs from asthmatic, atopic and healthy individuals were compared, we found no CpG sites that were differentially methylated between the subject groups (Figure 4D, E and F).

### Correlation between DNA Methylation and Gene Expression in AECs

To determine the impact of DNA methylation on gene expression in AECs, we chose to examine two genes. *CRIP1* as it had the most significant q-value and z-score difference for DNA methylation between atopic and asthmatic AECs (Table 3) and *STAT5A* because it had a significant q-value but was also identified a unique methylation CpG site in the analysis between asthmatic AECs verses asthmatic PBMCs (Table S5). We found that STAT5A gene expression was significantly reduced in AECS from asthmatic when compared to atopic and healthy subjects (Figure 5A). These data strongly support our findings of higher DNA methylation for the STAT5A_E42_F CpG site in asthmatic compared to atopic derived AECs (Figure 5B). Similar to STAT5A_E42_F, the CRIP1_P874_R CpG site had higher methylation in AECS from asthmatic compared to atopic subjects (Figure 5D). However, in contrast to STAT5A gene expression, CRIP1 gene expression was elevated in AECs from asthmatic when compared to both healthy and atopic subjects (Figure 5C).

## Discussion

In the current study, we characterized the methylation status of over 1000 CpG sites in AECs and PBMCs obtained from asthmatic, atopic and healthy children. We demonstrate a signature set of 57 CpG sites that are differentially methylated in AECs as compared to PBMCs regardless of disease phenotype. Our findings confirm that DNA methylation plays a role in tissue or cell specialization. We also identified 8 genes with differentially methylated CpG sites in AECs derived from asthmatic compared to AECs obtained from atopic children. Although gene markers in PBMCs have been identified through gene expression analysis for multiple diseases including aspirin-exacerbated respiratory disease and asthma [35], [36], we did not identify any differences in DNA methylation when comparing PBMCs from the three subject groups.

Characterization of DNA methylation profiles has been performed on epithelium derived from several tissues, such as ovary, prostate, breast and lung but these studies have been focused on cancer pathogenesis and cancer derived epithelial cell lines [37], [38], [39], [40], [41]. We identified 80 CpG sites in 67 genes which were differentially methylated in AECs compared to PBMCs. By analyzing this AEC DNA methylation signature by disease, we identified 57 CpG sites in 47 genes that were differentially methylated in AECs compared to PBMCs irrespective of disease status. These genes were identified by pathways analysis to be important in several cellular functions including cell cycle, cell signaling and cell metabolism. Such cell specific patterns are important in understanding the role of DNA methylation in cellular function and disease. Many of the CpG methylation differences we identified between AECs and PBMCs were to be expected as many are within the promoter region or exon 1 of genes that are specific for specialized cell functions. For example, we demonstrate that cytokeratin 5 (*KRT5*), a cytoskeletal protein, is less methylated in AECs compared to PBMCs (Table S1and S6). *KRT5* is a well documented marker of basal epithelial cells, it was therefore to be expected that this epithelial protein is repressed, and thus more methylated, in PBMCs. In contrast, *CD2*, a cell adhesion molecule expressed in certain lymphocytes, is less methylated in PBMCs compared to AECs (Table S1 and S6). These findings support the notion that DNA methylation is an important regulator of cell or tissue functions through the regulation of gene expression as previously described [42], [43]. In support of our findings is a report by Yang et al of a cell specific DNA methylation profile for a disintegrin and metalloprotease 33 (*ADAM33*) [44]. DNA methylation analysis of the promoter region revealed hypermethylation of *ADAM33* in epithelium and hypomethylation in fibroblasts which strictly regulated gene expression [44]. In addition, using a murine model, Hollingsworth et al identified an association between *in utero* supplementation with methyl donors and both allergic airways disease as well as differential methylation of the runt-related transcription factor 3 (*Runx3*) gene [4]. In our study, we identified elevated methylation of the *Runx3* gene at three separate CpG loci when we compared AECs to PBMCs from healthy subjects (Figure 3A, Table S3) but only two CpG sites in atopic and asthmatic subjects (Figure 3B and 3C, Table S4 and S5). Similarly, we identified differential methylation in the apolipoprotein A-1 (*apoA-1*) gene; we found decreased methylation in AECs as compared to PBMCs from all of our subjects (Figure 2B, Table S1) but, when we then compared AECs to PBMCs by phenotype, we found decreased methylation of *apoA-1* only in the asthmatic and atopic individuals (Figure 3B and 3C, Table S4 and S5). *ApoA-1* has recently been identified as a potential new therapeutic target for airway inflammation in asthma [45]. Using an apoA-1 mimetic peptide, Yao et al. were able to inhibit airway inflammation and hyperreactivity and attenuate manifestations of airway remodeling in a murine asthma model [45]. The alterations in methylation of *Runx3* and *apoA-1* which we identified in atopic and asthmatic subjects may well indicate a role for these genes in airway inflammation, however further validation is required.

We also identified differentially methylated CpG sites which were unique to each phenotype. Of interest, of the 6 unique differentially methylated CpG sites identified in asthmatic derived AECs compared to PBMCs (Figure 3D), one of these was the STAT5A_E42_F CpG site which we also found to be differentially methylated in asthmatic compared to atopic derived AECs, potentially highlighting the importance of this gene. Comparison of DNA methylation between AECs from atopic and asthmatic subjects yielded 8 genes that were differentially methylated of which the most significant was *CRIP1*. Our data suggest that atopy results from an epigenetically different profile from asthma rather than an intermediate phenotype. Support for this notion has come from GWAS studies in which some of the major asthma associated genes are epithelial in origin and do not segregate with allergy [46]. The effect of DNA methylation on STA5A and CRIP1 gene expression by disease status was further investigated. It is worth noting that we only found differential methylation based on atopy in AECs but not PBMCs, which are the source of monocytes associated with atopy and allergic inflammation (eosinophils, basophils, mast cells, and Th2 cells). As reported by Southam *et al*., in an allergic asthma murine model it has been shown that CD34^+^45^+^IL-5Rα^+^ cells, which are thought to be the earliest eosinophil lineage committed progenitor cell, give rise to eosinophil colonies only when isolated from the lungs of allergic but not control mice [47]. These data suggest that changes in local eosinophil numbers after allergen challenge may be caused, at least in part, by local lineage commitment and subsequent differentiation of progenitor cells via an IL-5 dependant mechanism. In future studies comparisons of tissue resident monocytes would potentially reveal epigenetic modifications in allergic subjects compared to non-atopic subjects.

*STAT5A* is a member of the STAT family of transcription factors, and is activated by a variety of cytokines and hormones. When activated by IL2, IL7, or TSLP, STAT5A is a strong promoter of Th2 cell differentiation and response [48], [49]. In an asthmatic mouse model, STAT5 phosphorylation was found to be elevated in OVA-induced splenocytes [50]. In mammary epithelial cells, activated STAT5 is required for tissue-specific gene expression via histone acetylation and chromatin remodeling of gene specific loci [51], while in the airway, STAT5 can be activated by Neuregulin-1, resulting in epithelial cell proliferation [52]. *STAT5A* knock-out mice displayed enhanced Th1 responses as well as decreased airway eosinophil recruitment [53]. In this study we demonstrate that, in asthmatic compared to both healthy and atopic derived AECs, STAT5A gene expression is decreased which is concordant with our finding of increased *STAT5A* methylation (Figure 5C and 5D). These data promote the notion that DNA methylation is a regulator of STAT5A gene expression in AECs. Based on the previous work on *STAT5A*, we would anticipate higher expression of this transcription factor in asthmatic derived AECs, however since we did not perform experiments measuring STAT5A activation, which could potentially modulate its effects, it remains to be seen how gene expression due to DNA methylation interacts with the cellular cytokine and hormonal milieu to impact STAT5A function. Therefore, altered DNA methylation in the *STAT5A* gene could have implications in allergic airways disease but more studies are necessary to elucidate the precise role of *STAT5A* in epithelial functions.

Due to its double zinc-finger motifs (LIM domains), *CRIP1* is involved in many cellular processes including motility, adhesion, and structure via its interaction with the cytoskeletal protein actin [54]. CRIP1 can also shuttle to the nucleus where it can facilitate protein interactions important for transcriptional regulation [55], [56]. In a transgenic mouse model, CRIP1 over-expression impacted host defense by skewing towards a Th2 phenotype as well as increasing host susceptibility to toxins from pathogens and viral infection [57]. Within PBMCs, CRIP1 has previously been identified as playing a role in the acute-phase immune response [58] but within epithelial cells its function is not as clear. In fibroblasts, CRIP1 is important in stress response being induced by growth-inhibitory signals and cytotoxic stress resulting in suppression of cell death and proliferation while elevating cellular attachment and metabolic activity [59]. Despite that lack of concordance between DNA methylation and gene expression, our findings of increased CRIP1 gene expression in the epithelium of asthmatics provides us with a new potential candidate gene that could be involved in many important epithelial functions. As the Illumina GoldenGate array focuses on CpG sites within the promoter region and or exon 1 of genes and not the gene body, methylation is likely to influence gene expression. However, it has been reported that DNA methylation can be overruled by histone modifications resulting in expression of a gene even if it is methylated and vise verse repress a gene when it is not methylated [60], [61], [62], [63]. In addition, agglomerative epigenetic aberrations including hypermethylation and hypomethylation have been described in several cancer studies [64], [65], [66], [67], [68]. These events affect large chromosomal regions where clusters of genes are silenced or activated as a result of a global epigenetic state. In long-range epigenetic silencing (LRES), although the majority of genes spanning these zones are hypermethylated, all of the genes in the region are transcriptionally repressed whether their promoters are methylated or not [66], [69]. Thus, it is possible that regulation of CRIP1 gene expression is more complex, potentially involving other factors such as histone modifications and global epigenetic states.

Asthma is classically characterized by chronic airway inflammation, variable airway obstruction, airway remodeling and hyperresponsiveness [70]. Allergic asthma involves the adaptive immune response which is triggered upon allergen exposure resulting in a T-helper type 2 (Th2) biased inflammatory response [71]. In contrast, the mechanisms involved in non-allergic asthma are less well defined and are thought to involve an innate immune response that may be activated by environmental factors such as air pollution or oxidative stress. We thus wanted to identify if differential epigenetic hallmarks could be found between these two asthma phenotypes, yet we found that PBMCs or AECs from non-atopic and atopic asthmatics shared the same epigenetic signature across the 1027 CpG sites examined. Therefore, even though our sample size was small, this suggests that the biological processes involved in these two asthma phenotypes are not influenced by DNA methylation of the genes examined in this study thus we grouped the asthmatic subjects as one phenotype and used this for all subsequent analyses.

This study has several limitations. While a small sample size for each disease phenotype studied is likely to influence our ability to detect differential CpG methylation, we were able to determine a number of differentially methylated CpG sites between asthmatic and atopic AECs. Additionally, we used the Illumina GoldenGate Methylation Cancer Panel I which was designed to screen candidate genes originally identified in cancers and thus focuses on cancer related pathways. Even so, we were able to identify pathways within this cancer-centric structure that were relevant to our study. The inability to determine the entire epigenetic profile of the airway epithelium may result in potentially missing many disease specific epigenetic modifications. With regards to disease, our study also focused on pediatric subjects with asthma and atopy. Therefore we cannot determine the epigenetic component of atopy and asthma with increasing age, which may identify a stronger epigenetic fingerprint. Also, all subjects included in the study displayed mild disease therefore it is difficult to generalize our results to all subtypes and severities of asthma. However our findings do indicate that epigenetic changes are present early in disease pathogenesis and highlight the importance of understanding these mechanisms of gene regulation further. Although the individuals within the study were well characterized for asthma and atopy we did not have enough power in the study cohort to stratify the groups by specific allergens, which may also influence the epigenetic profile of the airway epithelium.

In summary, we have characterized a cell specific DNA methylation signature for AECs and PBMCs that is maintained regardless of the presence of asthma or atopy. Our data also highlights the importance of determining the effects of DNA methylation in the airway epithelium in discriminating atopy and asthma rather than using PBMCs. We conclude that future studies are required to determine the complete epigenetic signature of the airway epithelium in individuals with atopy and asthma to elucidate candidate genes which may be involved in the pathogenesis of disease and amenable to targeted therapies.

## Supporting Information

Table S1 **Differentially Methylated CpG Sites in AECs Compared to PBMCs.** We identified 80 CpG sites which are differentially methylated between AECs and PBMCs. Z-score difference is presented as AEC relative to PBMC.
(DOCX)

Table S2 **Biological Functions in Differentially Methylated Genes in AECs Compared to PBMCs.** We identified 67 genes which contain the 80 differentially methylated CpG sites between AECs and PBMCs, these genes are classified into 19 overrepresented biological functions.
(DOCX)

Table S3 **Differentially Methylated Sites in Healthy AECs Compared to PBMCs.** We identified 96 CpG sites which are differentially methylated between healthy AECs and PBMCs. Z-score difference is presented as AECs relative to PBMCs.
(DOCX)

Table S4 **Differentially Methylated Sites in Atopic AECs Compared to PBMCs.** We identified 71 CpG sites which are differentially methylated between atopic AECs and PBMCs. Z-score difference is presented as AECs relative to PBMCs.
(DOCX)

Table S5 **Differentially Methylated Sites in Asthmatic AECs Compared to PBMCs.** We identified 89 CpG sites which are differentially methylated between asthmatic AECs and PBMCs. Z-score difference is presented as AECs relative to PBMCs.
(DOCX)

Table S6 **Biological Functions in Differentially Methylated Core Genes in AECs Compared to PBMCs.** We identified 47 core genes which contain 57 differentially methylated CpG sites between AECs and PBMCs, these genes are classified into 19 overrepresented biological functions.
(DOCX)
