# Supplementary material for: DNA Methylation Profiles of Airway Epithelial Cells and PBMCs from Healthy, Atopic and Asthmatic Children
Source: PLoS One. 2012 Sep 6;7(9):e44213. doi: 10.1371/journal.pone.0044213 (PMC3435400; doi:10.1371/journal.pone.0044213)
Supplement: Table S1 — Differentially Methylated CpG Sites in AECs Compared to PBMCs. We identified 80 CpG sites which are differentially methylated between AECs and PBMCs. Z-score difference is presented as AEC relative to PBMC. (DOCX) [file pone.0044213.s001.docx]

**Table S1. Differentially Methylated CpG Sites in AECs Compared to PBMCs.**

| **CpG Site** | **z-score difference (log2)** | **q-value** |
| --- | --- | --- |
| S100A2_P1186_F | -2.03 | <0.001 |
| FRK_P36_F | -1.91 | <0.001 |
| TRIM29_E189_F | -1.89 | <0.001 |
| DDR1_P332_R | -1.83 | <0.001 |
| RARRES1_P426_R | -1.78 | <0.001 |
| NBL1_P24_F | -1.76 | <0.001 |
| LY6G6E_P45_R | -1.56 | <0.001 |
| HOXA5_E187_F | -1.46 | <0.001 |
| HOXA5_P479_F | -1.45 | <0.001 |
| SERPINB5_P19_R | -1.41 | <0.001 |
| NID1_P677_F | -1.41 | <0.001 |
| KRT5_E196_R | -1.40 | <0.001 |
| LIG3_P622_R | -1.40 | <0.001 |
| IL2_P607_R | -1.40 | <0.001 |
| SFN_E118_F | -1.39 | <0.001 |
| TGFB3_E58_R | -1.38 | <0.001 |
| ACVR1_P983_F | -1.33 | <0.001 |
| TRIM29_P135_F | -1.30 | <0.001 |
| TRIP6_P1090_F | -1.30 | <0.001 |
| SNCG_P53_F | -1.29 | <0.001 |
| MC2R_P1025_F | -1.27 | <0.001 |
| SPDEF_P6_R | -1.25 | <0.001 |
| TMPRSS4_E83_F | -1.25 | <0.001 |
| NOS3_P38_F | -1.24 | <0.001 |
| KLK11_P103_R | -1.24 | <0.001 |
| PRSS8_E134_R | -1.21 | <0.001 |
| FGF1_E5_F | -1.21 | <0.001 |
| MET_E333_F | -1.18 | <0.001 |
| SNCG_E119_F | -1.13 | <0.001 |
| GLI2_P295_F | -1.11 | <0.001 |
| PLAT_P80_F | -1.10 | <0.001 |
| CXCL9_E268_R | -1.09 | <0.001 |
| PTK6_E50_F | -1.09 | <0.001 |
| CTGF_P693_R | -1.09 | <0.001 |
| MST1R_P87_R | -1.05 | <0.001 |
| APOA1_P261_F | -1.04 | <0.001 |
| S100A2_E36_R | -1.03 | <0.001 |
| RIPK1_P868_F | -1.03 | <0.001 |
| FGF1_P357_R | -1.01 | <0.001 |
| RUNX3_E27_R | 1.01 | <0.001 |
| TIE1_E66_R | 1.05 | <0.001 |
| CRIP1_P874_R | 1.14 | <0.001 |
| LTA_P214_R | 1.14 | <0.001 |
| TM7SF3_P1068_R | 1.16 | <0.001 |
| PPARG_P693_F | 1.17 | <0.001 |
| DLC1_E276_F | 1.17 | <0.001 |
| MT1A_P600_F | 1.19 | <0.001 |
| SLC22A3_P634_F | 1.19 | <0.001 |
| PECAM1_P135_F | 1.22 | <0.001 |
| ICAM1_P386_R | 1.23 | <0.001 |
| ERCC3_P1210_R | 1.24 | <0.001 |
| TBX1_P520_F | 1.25 | <0.001 |
| PSCA_E359_F | 1.27 | <0.001 |
| RUNX3_P247_F | 1.27 | <0.001 |
| RUNX3_P393_R | 1.30 | <0.001 |
| MPL_P62_F | 1.31 | <0.001 |
| S100A4_P194_R | 1.33 | <0.001 |
| AIM2_P624_F | 1.37 | <0.001 |
| SPI1_P48_F | 1.38 | <0.001 |
| CD34_P339_R | 1.42 | <0.001 |
| LAT_E46_F | 1.44 | <0.001 |
| GP1BB_E23_F | 1.47 | <0.001 |
| CD86_P3_F | 1.51 | <0.001 |
| IL10_P348_F | 1.53 | <0.001 |
| EVI2A_E420_F | 1.53 | <0.001 |
| OSM_P188_F | 1.56 | <0.001 |
| RARA_P1076_R | 1.56 | <0.001 |
| LTB4R_E64_R | 1.59 | <0.001 |
| PADI4_E24_F | 1.62 | <0.001 |
| OSM_P34_F | 1.66 | <0.001 |
| CD34_P780_R | 1.68 | <0.001 |
| LMO2_E148_F | 1.75 | <0.001 |
| RAB32_P493_R | 1.76 | <0.001 |
| LTB4R_P163_F | 1.81 | <0.001 |
| CD2_P68_F | 2.09 | <0.001 |
| TBX1_P885_R | 2.20 | <0.001 |
| TNFSF8_P184_F | 2.22 | <0.001 |
| AFF3_P122_F | 2.36 | <0.001 |
| TNFSF8_E258_R | 2.87 | <0.001 |
| GP1BB_P278_R | 3.08 | <0.001 |
