# Supplementary material for: DNA Methylation Profiles of Airway Epithelial Cells and PBMCs from Healthy, Atopic and Asthmatic Children
Source: PLoS One. 2012 Sep 6;7(9):e44213. doi: 10.1371/journal.pone.0044213 (PMC3435400; doi:10.1371/journal.pone.0044213)
Supplement: Table S2 — Biological Functions in Differentially Methylated Genes in AECs Compared to PBMCs. We identified 67 genes which contain the 80 differentially methylated CpG sites between AECs and PBMCs, these genes are classified into 19 overrepresented biological functions. (DOCX) [file pone.0044213.s002.docx]

**Table S2.** **Biological Functions in Differentially Methylated Genes in AECs Compared to PBMCs.**

| **Category** | **Genes** |
| --- | --- |
| Cell-To-Cell Signaling and Interaction | PPARG, CTGF, ICAM1, CXCL9, CD2, IL10, APOA1, S100A4, PSCA, NOS3, SPI1, DDR1, FGF1, TNFSF8, LTB4R, IL2, LTA, LAT, CD86, OSM, PECAM1, SERPINB5, CD34 |
| Cell Death | PPARG, RUNX3, ICAM1, CD2, IL10, DLC1, SPI1, TNFSF8, RIPK1, IL2, LTA, ERCC3, TGFB3, OSM |
| Cellular Movement | CTGF, ICAM1, GLI2, CD2, APOA1, S100A4, RARRES1, NOS3, SPDEF, SPI1, LTB4R, IL2, RARA, OSM, SERPINB5, PLAT, PPARG, RUNX3, CXCL9, NBL1, IL10, ACVR1, TRIP6, SNCG, DLC1, S100A2, DDR1, TIE1, FGF1, MET, LTA, TGFB3, PTK6, CD86, PECAM1, MST1R, TBX1, CD34 |
| Antigen Presentation | RUNX3, CXCL9, ICAM1, CD2, IL2, APOA1, IL10, CD86, SPI1 |
| Cellular Compromise | CD2,IL2,S100A4,CD86 |
| Cellular Development | PPARG, RUNX3, ICAM1, CD2, APOA1, IL10, LMO2, SPI1, DDR1, FGF1, TNFSF8, MET, MPL, IL2, LTA, RARA, LAT, HOXA5, OSM, PECAM1, CD86 |
| Cellular Growth and Proliferation | PPARG, LTB4R, ICAM1, CD2, IL2, IL10, LTA, RARA, OSM, CD86, SPI1 |
| Gene Expression | PPARG, RUNX3, GLI2, CD2, IL10, LMO2, SPDEF, SPI1, FGF1, TNFSF8, MET, RIPK1, IL2, ERCC3, RARA, HOXA5, TGFB3, OSM, TRIM29, TBX1 |
| Protein Synthesis | PPARG, APOA1, IL10, IL2, LTA, CD86, SFN, NOS3, SPI1 |
| Carbohydrate Metabolism | IL2, IL10, APOA1, OSM, FGF1 |
| Cell Morphology | MPL, CD2, IL2, IL10, LAT, CD86, GP1BB, SPI1 |
| Cellular Function and Maintenance | PPARG, ICAM1, IL10, IL2, LMO2, LAT, CD86, SPI1 |
| Molecular Transport | APOA1, IL10, NOS3, MC2R |
| Cell Signaling | ICAM1, IL10, RARA, NOS3, TBX1 |
| Small Molecule Biochemistry | ICAM1, IL2, IL10, OSM, NOS3, CD34, FGF1 |
| Amino Acid Metabolism | NOS3, CD34 |
| Cell Cycle | PPARG, PADI4 |
| Cellular Assembly and Organization | CD2, IL10, LAT, CD86, MST1R |
| DNA Replication, Recombination and Repair | IL10, MST1R |
