# Supplementary material for: DNA Methylation Profiles of Airway Epithelial Cells and PBMCs from Healthy, Atopic and Asthmatic Children
Source: PLoS One. 2012 Sep 6;7(9):e44213. doi: 10.1371/journal.pone.0044213 (PMC3435400; doi:10.1371/journal.pone.0044213)
Supplement: Table S3 — Differentially Methylated Sites in Healthy AECs Compared to PBMCs. We identified 96 CpG sites which are differentially methylated between healthy AECs and PBMCs. Z-score difference is presented as AECs relative to PBMCs. (DOCX) [file pone.0044213.s003.docx]

**Table S3.** **Differentially Methylated Sites in Healthy AECs Compared to PBMCs.**

.

| **CpG Site** | **z-score difference (log2)** | **q-value** |
| --- | --- | --- |
| TRIM29_E189_F | -2.12 | <0.001 |
| S100A2_P1186_F | -2.09 | <0.001 |
| FRK_P36_F | -2.05 | <0.001 |
| DDR1_P332_R | -2.04 | <0.001 |
| NBL1_P24_F | -1.76 | <0.001 |
| LIG3_P622_R | -1.72 | <0.001 |
| RARRES1_P426_R | -1.72 | <0.001 |
| HOXA5_P479_F | -1.70 | <0.001 |
| LY6G6E_P45_R | -1.63 | 0.001 |
| KLK11_P103_R | -1.63 | <0.001 |
| TRIP6_P1090_F | -1.61 | <0.001 |
| CTGF_P693_R | -1.53 | 0.017 |
| SERPINB5_P19_R | -1.48 | <0.001 |
| MC2R_P1025_F | -1.44 | <0.001 |
| SFN_E118_F | -1.44 | <0.001 |
| HOXA5_E187_F | -1.40 | <0.001 |
| GLI2_P295_F | -1.38 | <0.001 |
| KRT5_E196_R | -1.37 | <0.001 |
| TRIM29_P135_F | -1.37 | <0.001 |
| ACVR1_P983_F | -1.32 | <0.001 |
| SNCG_E119_F | -1.32 | <0.001 |
| IL2_P607_R | -1.32 | <0.001 |
| CXCL9_E268_R | -1.32 | <0.001 |
| SNCG_P53_F | -1.29 | <0.001 |
| NPR2_P618_F | -1.26 | <0.001 |
| NID1_P677_F | -1.26 | <0.001 |
| HHIP_P578_R | -1.25 | <0.001 |
| MET_E333_F | -1.25 | <0.001 |
| NOS3_P38_F | -1.25 | 0.001 |
| CSF3_E242_R | -1.24 | <0.001 |
| SPDEF_P6_R | -1.24 | <0.001 |
| LCN2_P141_R | -1.20 | <0.001 |
| PRSS8_E134_R | -1.20 | <0.001 |
| EPHA2_P340_R | -1.19 | <0.001 |
| TMPRSS4_E83_F | -1.18 | <0.001 |
| PTK6_E50_F | -1.15 | <0.001 |
| TGFB3_E58_R | -1.14 | <0.001 |
| GRB7_E71_R | -1.13 | <0.001 |
| ZIM3_P451_R | -1.10 | <0.001 |
| PLAT_P80_F | -1.10 | <0.001 |
| IGFBP5_P9_R | -1.10 | <0.001 |
| RIPK1_P868_F | -1.10 | <0.001 |
| S100A2_E36_R | -1.08 | <0.001 |
| CALCA_E174_R | -1.08 | <0.001 |
| NOS2A_E117_R | -1.08 | <0.001 |
| MST1R_P87_R | -1.07 | <0.001 |
| CARD15_P302_R | -1.06 | <0.001 |
| FGF1_E5_F | -1.05 | <0.001 |
| FASTK_P598_R | -1.05 | <0.001 |
| DSC2_E90_F | -1.04 | <0.001 |
| MMP14_P208_R | -1.03 | <0.001 |
| ERG_E28_F | -1.02 | <0.001 |
| CD82_P557_R | -1.02 | <0.001 |
| DLC1_P695_F | 1.10 | 0.013 |
| MT1A_P600_F | 1.13 | <0.001 |
| MPL_P62_F | 1.13 | <0.001 |
| JAK2_P772_R | 1.16 | <0.001 |
| ITK_E166_R | 1.19 | <0.001 |
| LTA_P214_R | 1.19 | <0.001 |
| ERCC3_P1210_R | 1.19 | <0.001 |
| TBX1_P520_F | 1.21 | <0.001 |
| PECAM1_P135_F | 1.24 | <0.001 |
| SPI1_P48_F | 1.26 | <0.001 |
| CD34_P339_R | 1.29 | <0.001 |
| TM7SF3_P1068_R | 1.29 | <0.001 |
| RUNX3_E27_R | 1.31 | <0.001 |
| CRIP1_P874_R | 1.32 | 0.002 |
| ICAM1_P386_R | 1.33 | 0.001 |
| PSCA_E359_F | 1.40 | <0.001 |
| S100A4_P194_R | 1.40 | <0.001 |
| IL10_P348_F | 1.40 | <0.001 |
| DLC1_E276_F | 1.42 | <0.001 |
| AIM2_P624_F | 1.42 | <0.001 |
| SLC22A3_P634_F | 1.47 | 0.002 |
| PADI4_E24_F | 1.48 | <0.001 |
| RUNX3_P393_R | 1.51 | <0.001 |
| LAT_E46_F | 1.51 | <0.001 |
| PPARG_P693_F | 1.52 | <0.001 |
| OSM_P34_F | 1.52 | <0.001 |
| RARA_P1076_R | 1.53 | <0.001 |
| RUNX3_P247_F | 1.56 | <0.001 |
| EVI2A_E420_F | 1.57 | <0.001 |
| OSM_P188_F | 1.58 | <0.001 |
| GP1BB_E23_F | 1.60 | <0.001 |
| LTB4R_E64_R | 1.60 | <0.001 |
| CD86_P3_F | 1.76 | <0.001 |
| CD2_P68_F | 1.80 | <0.001 |
| LMO2_E148_F | 1.81 | <0.001 |
| RAB32_P493_R | 1.82 | <0.001 |
| CD34_P780_R | 1.82 | <0.001 |
| LTB4R_P163_F | 1.88 | <0.001 |
| TNFSF8_P184_F | 1.92 | <0.001 |
| AFF3_P122_F | 2.05 | <0.001 |
| TBX1_P885_R | 2.51 | <0.001 |
| TNFSF8_E258_R | 2.94 | <0.001 |
| GP1BB_P278_R | 3.16 | <0.001 |
