# Supplementary material for: DNA Methylation Profiles of Airway Epithelial Cells and PBMCs from Healthy, Atopic and Asthmatic Children
Source: PLoS One. 2012 Sep 6;7(9):e44213. doi: 10.1371/journal.pone.0044213 (PMC3435400; doi:10.1371/journal.pone.0044213)
Supplement: Table S6 — Biological Functions in Differentially Methylated Core Genes in AECs Compared to PBMCs. We identified 47 core genes which contain 57 differentially methylated CpG sites between AECs and PBMCs, these genes are classified into 19 overrepresented biological functions. (DOCX) [file pone.0044213.s006.docx]

**Table S6.**  **Biological Functions in Differentially Methylated Core Genes in AECs Compared to PBMCs.**

| **Category** | **Genes** |
| --- | --- |
| Cell-To-Cell Signaling and Interaction | ICAM1, CD2, IL10, SPI1, DDR1, FGF1, TNFSF8, LTB4R, IL2, LTA, LAT, CD86, OSM, CD34 |
| Cellular Development | RUNX3, ICAM1, CD2, IL10, LMO2, SPI1, DDR1, FGF1, TNFSF8, MPL, IL2, LTA, RARA, LAT, HOXA5, TGFB3, OSM, CD86, SFN, CD34 |
| Cellular Compromise | CD2, IL2, S100A4, CD86 |
| Cellular Growth and Proliferation | RUNX3, ICAM1, CD2, IL10, SPI1, FGF1, LTB4R, IL2, RARA, LTA, TGFB3, CD86, OSM |
| Cell Death | RUNX3, ICAM1, CD2, IL10, SPI1, TNFSF8, IL2, LTA, ERCC3, LAT, TGFB3, OSM, CD86 |
| Gene Expression | RUNX3, CD2, IL10, LMO2, SPI1, FGF1, TNFSF8, IL2, ERCC3, RARA, HOXA5, TGFB3, OSM, TRIM29, TBX1 |
| Protein Synthesis | IL2, IL10 |
| Cell Morphology | RUNX3, ICAM1, CD2, IL10, SPI1, MPL, IL2, LTA, LAT, HOXA5, CD86, GP1BB, CD34 |
| Cellular Function and Maintenance | TNFSF8, RUNX3, ICAM1, CD2, IL10, IL2, LMO2, LTA, LAT, CD86, OSM, SPI1 |
| Cellular Movement | RUNX3, ICAM1, CD2, IL2, IL10, LTA, S100A4, OSM, CD86 |
| Carbohydrate Metabolism | IL2, IL10, OSM, FGF1 |
| Small Molecule Biochemistry | IL2, IL10, OSM, SNCG, SFN, FGF1 |
| Antigen Presentation | ICAM1, CD2, IL2, IL10, SPI1 |
| Cell Signaling | RARA, TBX1 |
| Cellular Assembly and Organization | CD2, LAT, CD86 |
| Drug Metabolism | IL2, IL10, OSM, SNCG, SFN |
| Molecular Transport | ICAM1, CD2, IL2, IL10, LTA, LAT, S100A4, OSM, MT1A, MC2R, FGF1 |
| Lipid Metabolism | SNCG, SFN |
| Cell Cycle | OSM, FGF1 |
